# Learning curve of consolers and bedside surgeons fused robotic-assisted thoracoscopic segmentectomy: insights from the initial 100 cases

**DOI:** 10.1007/s00595-024-02957-0

**Published:** 2024-11-14

**Authors:** Yuki Uno, Shinya Tane, Yugo Tanaka, Midori Takanashi, Takefumi Doi, Hiroyuki Ogawa, Daisuke Hokka, Yoshimasa Maniwa

**Affiliations:** Division of Thoracic Surgery, Kobe University Graduate School of Medicine, 7-5-2, Kusunoki-Cho, Chuo-Ward, Kobe, 650-0017 Japan

**Keywords:** Robot-assisted thoracoscopic surgery, Segmentectomy, Learning curve

## Abstract

**Purpose:**

This study examined the learning curve of segmentectomy using the “fused surgery” approach.

**Methods:**

We retrospectively collected data from 100 patients who underwent segmentectomy via fused robot-assisted thoracoscopy at our institution between September 2020 and February 2024. The learning curve was evaluated using the cumulative sum of the operative times in all cases and was analyzed separately for simple and complex segmentectomies.

**Results:**

After applying the cumulative sum method to all cases, we obtained a graph of the operative time that showed three well-differentiated phases: phase 1 (*n* = 23), the initial learning phase; phase 2 (*n* = 28), the increased competence phase; and phase 3 (*n* = 49), the highest skill phase. Comparing phases 1 and 2 with phase 3, we found significant differences in operative time (*P* < 0.001); however, no significant differences were observed in bleeding or rate of postoperative complications. We observed a significant reduction in operative time after 25 simple segmentectomies and 22 complex segmentectomies.

**Conclusions:**

The data suggested that the inflection point of the learning curve was achieved in 51 cases. Complex segmentectomy requires the same cases to achieve the same level of competence as simple segmentectomy.

## Introduction

Minimally invasive techniques have revolutionized thoracic surgery, and robotic approaches are being used increasingly frequently. Some studies have been published on the advantages of robot-assisted thoracoscopic surgery (RATS), which allows a three-dimensional view with a resolution of up to 10 × and accurate and amplified movement articulation [1, 2]. However, longer operative times were observed in RATS than in video-assisted thoracoscopic surgery, possibly because of a lack of familiarity with robotic surgery, such as docking, troubleshooting, and port placement [3–5]. Therefore, gaining experience in performing the procedure has been suggested to reduce operative time.

Since the introduction of the robotic approach in 2019, we have developed a” Fused Surgery’ approach, in which the consoler and bedside surgeons cooperate during each procedure [6, 7]. This approach offers educational and learning advantages. Bedside surgeons can use curved suction or a cotton swab to expand the operative view and a mechanical stapler or energy device to dissect the vasculature, which could help improve their surgical skills and efficiency. Our institution is an educational hospital; hence, the leading surgeon is responsible for teaching surgical techniques to the residents. This is important, even though robotic surgery is often characterized as “solo surgery” owing to its high-quality maneuverability and adaptability by the console surgeon.

Segmentectomy has gained popularity in the field of thoracic surgery and has been applied as an alternative to lobectomy [8, 9]. Therefore, to establish a straightforward robotic surgical team using fused RATS and standardize our technique for segmentectomy, analyzing the learning curve is indispensable. The cumulative sum (CUSUM) method was adopted in the 1970s to analyze learning curves for surgical procedures [10].

We herein report the results of our study evaluating the learning curve and our initial experience with segmentectomy using a fused RATS approach.

## Materials and methods

### Ethical statement

The Kobe University Hospital Institutional Review Board (IRB) approved the study (IRB number: B-240023; approved on March 12), and each participant provided their informed consent.

### Patient collection

This study reviewed and analyzed clinicopathological data and prognosis of 100 consecutive patients who underwent segmentectomy for primary lung cancer and pulmonary metastatic tumors between August 2020 and February 2024 at Kobe University Hospital.

### Procedure

RATS lobectomy was performed using a da Vinci X or Xi Surgical System (Intuitive Surgical, Sunnyvale, CA, USA). Since November 2021, both the X and Xi Surgical Systems have been used.

The patient was placed in the lateral recumbent position, and axillary pillows and bed flexion were used to secure the intercostal space and prevent interference between the robotic arms and pelvis. Four ports (including a camera port) each were placed in the 7th intercostal space in the upper right, lower right, and lower left lobes. Similarly, four more ports were similarly placed in the 7th intercostal space of the left upper lobe. In patients with slender builds, the dorsal port often moves to the caudal intercostal space where the ribs are more craniocaudally inclined. The patient-side surgeon operated the conventional automatic staplers and energy devices using a robotic port without a robotic arm or an access port in the 4th intercostal space for the upper lobe or the 5th intercostal space for lower lobe, respectively. Seven consolers and 18 bedside surgeons participated in this study. Consolers occasionally also served as bedside surgeons, sometimes in the capacity of teaching assistants to consolers.

First, the affected hilar structures were exposed, such as the segmental vessels and bronchus, which were dissected toward the periphery and subsequently severed. The intersegmental planes were identified by systemic injection of indocyanine green (0.3 mg/kg) using a near-infrared thoracoscopic camera (Firefly Fluorescence Imaging camera; Intuitive Surgical) to determine the demarcation line. The intersegmental planes were dissected using a staple device. To prevent prolonged air leakage, we used a bioabsorbable polyglycolic mesh in combination with fibrin glue immediately after the intraoperative detection. Selective mediastinal lymphadenectomy is generally performed for lymph node dissections. For compromised patients and those with pulmonary metastasis, intraoperative hilar or mediastinal node sampling was only performed when the nodes exhibited a preoperative ^18^F-fluorodeoxyglucose uptake. If a positive lymph node was suspected based on intraoperative visualization, pathologic node assessments were performed using frozen section analyses. When the lymph nodes submitted for frozen sections were positive, we converted segmentectomy to lobectomy.

Segmentectomy is categorized as simple or complex in terms of procedural difficulty. Simple segmentectomy included resection of the 6th segment, left upper division, lingula, and basal segments. Complex segmentectomy includes segmentectomies other than those categorized as simple segmentectomies, as described elsewhere [11].

### Analyzing the learning curve

The learning curve was analyzed using the CUSUM method, as described by Wohl et al. [10]. The CUSUM is the total difference between individual data points and the mean of all data points; thus, it can be performed recursively. The learning curve was evaluated using the operation time (OT) and cumulative sum value of operation time (CUSUM-OT) in all cases and groups for simple and complex segmentectomies. First, the cases were chronologically ordered from the earliest to latest surgery date. The CUSUM-OT in the first case was the difference between the operation time in the first case and the mean operation time in all cases. The CUSUM-OT of the second case was that of the previous case, which was added to the difference between the operative time for the second case and the mean operative time for all cases. This recursive process continued until CUSUM-OT for the last case was calculated as zero. The required cases were calculated from the inflection point of the CUSUM-OT curve, representing the best fit of the plot.

### Statistical analyses

The Mann–Whitney U test was used to compare continuous variables, and Fisher’s exact test was used to compare nominal variables. All statistical analyses were performed using JMP software (version 16; SAS Inc., Cary, NC, USA). All values, except the amount of bleeding and drainage period, are expressed as the mean ± standard deviation. Differences were considered statistically significant at *p* < 0.05.

## Results

During the study period, 100 patients underwent RATS. Patient demographics, surgical procedures, intraoperative characteristics, short-term postoperative outcomes, and pathological diagnoses are summarized in Table 1. Post-operative mortality was not observed in this population.

**Table 1:** Patients characteristics

Characteristics	Data (*n* = 100)
Age (years)	70.3 ± 0.8 (50–84)
Sex (Male/Female)	55/45
Diagnosis	
Lung cancer	82
Metastatic tumor	16
Others	2
Comorbidity	
Emphysema (%)	25 (25)
Interstitial pneumonia (%)	1 (1)
Disease	
Lung cancer stage (UICC ver8) 0	3
1A1	31
1A2	31
1A3	9
Metastatic pulmonary lesion	26
FEV1.0/FVC (%)	73.5 ± 9.0
Operation time (min)	184 ± 21
Amount of bleeding (ml)	45.3 ± 5.2
Resected segment	
Right (n = 43)	
S1	2
S2	6
S3	4
S6	15
S7	1
S8	6
S8 + 9	1
S9 + 10	5
S10	2
Basal	1
Left (n = 57)	
S1 + 2	4
S1 + 2 + 3	23
S4 + 5	4
S6	8
S8	6
S8 + 9	3
S9 + 10	3
Basal	6

Table 2 shows patient characteristics and surgical outcomes according to simple and complex segmentectomies. The OT of complex segmentectomy was significantly longer than that of simple segmentectomy (176 *vs*. 151 min, *p* = 0.002); however, postoperative complications, including prolonged air leakage, did not differ markedly between the two groups (17.7% *vs*. 10.9%, *p* = 0.33). We experienced only one conversion to VATS because of bleeding from the basal artery during left S6 segmentectomy.

**Table 2:** Patient characteristics and surgical outcome in simple and complex segmentectomy

	Simple segmentectomy (*n* = 55)	Complex segmentectomy (*n* = 45)	*P* value
Sex (male/female)	31/24	24/21	0.76
Age (years)	70.0 ± 8.7	69.0 ± 10.4	0.85
BMI (m^2^/kg)	22.3 ± 3.4	23.5 ± 3.8	0.11
Operation time (min)	151 ± 33.6	176 ± 43.9	0.002
Amount of bleeding (ml) (median, IQR)	5 (5–10)	5 (5–10)	0.62
Number of staples used	8 ± 1.77	9 ± 2.40	0.062
FEV1.0 (L)	2.27 ± 0.12	2.30 ± 0.09	0.59
FEV1.0/FVC (%)	72 ± 1	74 ± 1	0.74
Tumor size (cm)	17.2 ± 6.6	16.6 ± 7.5	0.66
Conversion	1 (1.8)	0 (0)	0.27
Drainage period (days) (median, IQR)	2 (2–3)	2 (2–5)	0.11
Complication (%)	6 (10.9)	8 (17.8)	0.33

*BMI* Body mass index, *FEV1.0* Forced expiratory volume in 1 s, *FVC* Forced vital capacity, *IQR* INTERQURTILE range

**Fig. 1:**
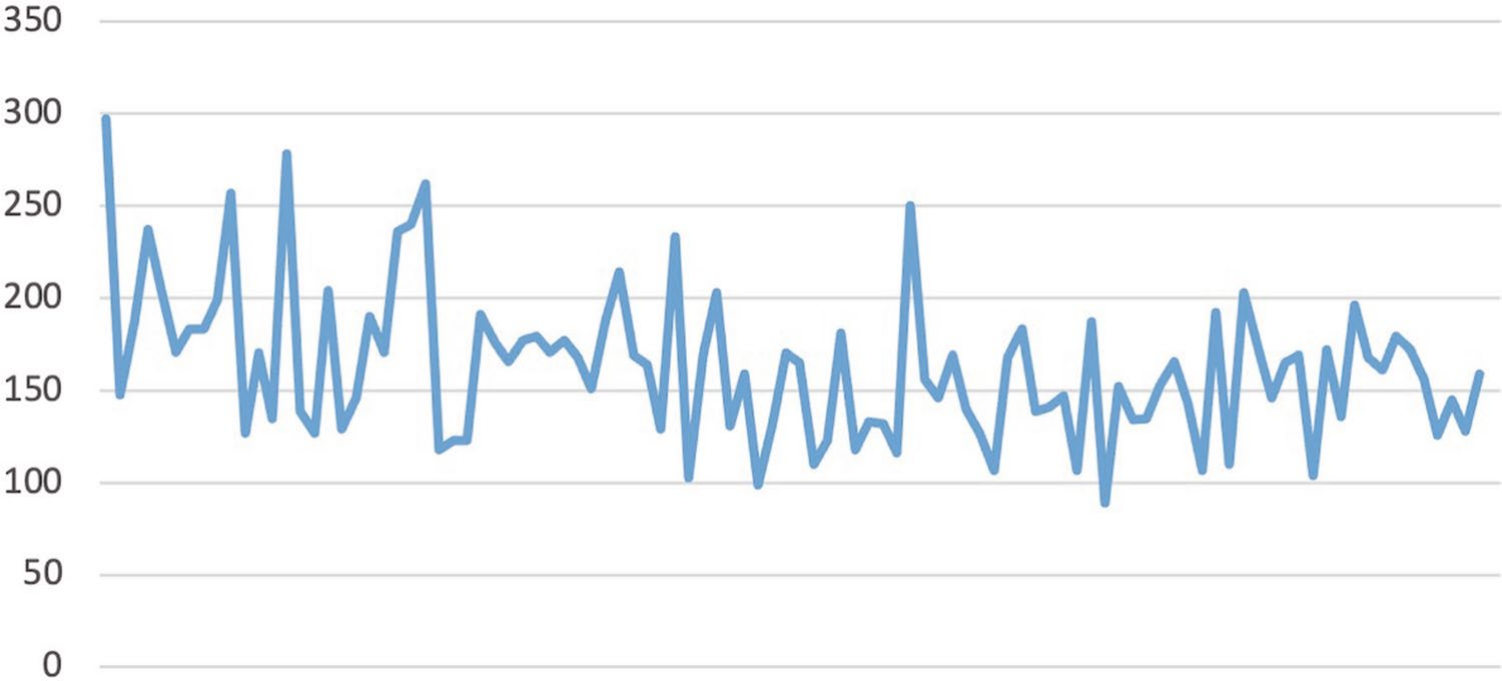
Graph of raw operative times plotted for each 100 consecutive patients

**Fig. 2:**
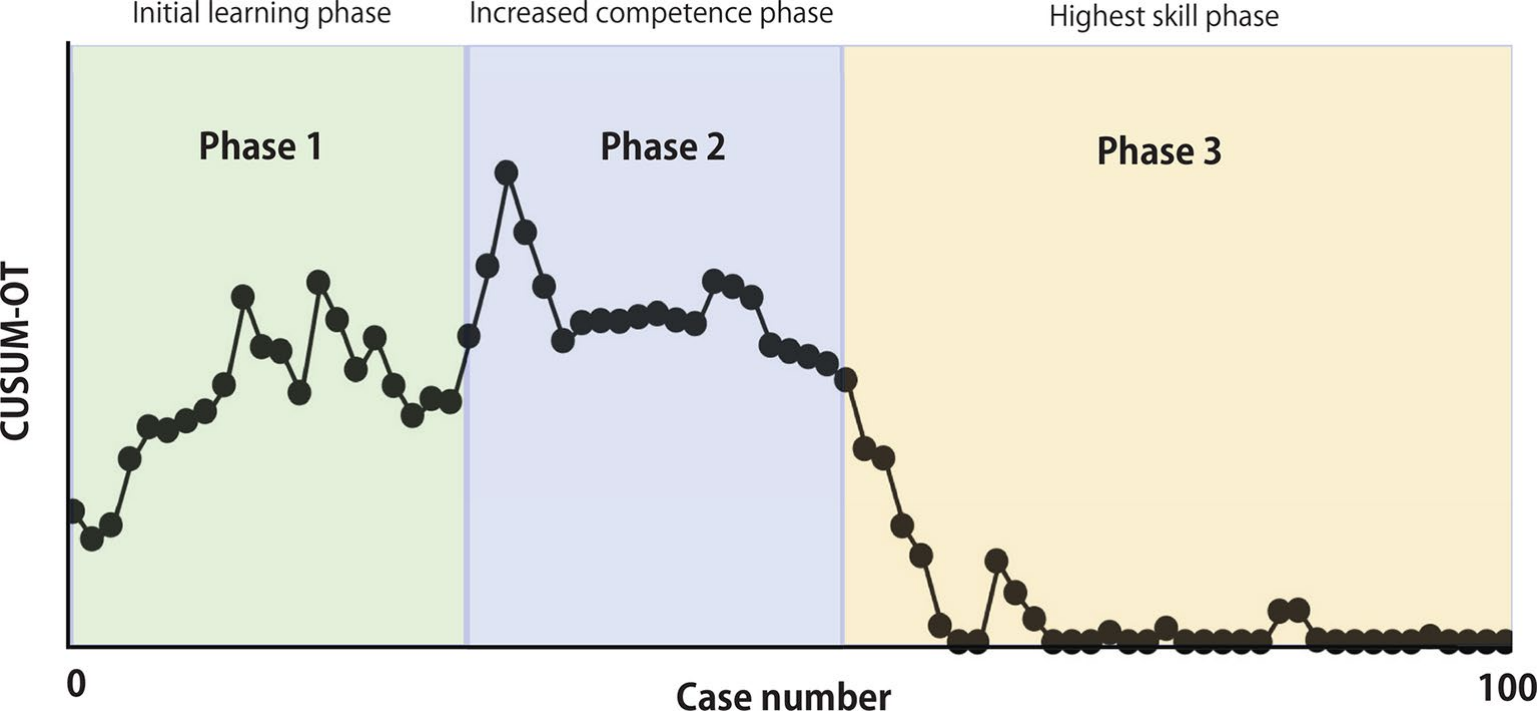
Three phases of operative time in terms of the CUSUM learning curve in the 100 consecutive patients CUSUM: cumulative sum

Figure 1 shows the raw OT in chronological order. Once OT was arranged, we calculated the CUSUM-OT values for each case to obtain a graph of the learning curve (Fig. 2). We differentiated three phases in the graph: Phase 1 (the initial learning phase), Cases 1–23; Phase 2 (the increased competence phase), Cases 24–51; and Phase 3 (the highest-skill phase), Cases 52–100. Inflection points were observed in 51 patients. Comparisons of the various parameters among the three phases identified by the CUSUM-OT analysis are presented in Table 3. The OT was significantly higher in the initial learning and increased competence phases (phase 1 + 2) than in the highest skill phase (phase 3) (*p* < 0.002). Although not significantly different, complications tended to be more frequent in the initial learning and increased competence phases (phase 1 + 2) than in the highest skill phase (phase 3) (19.6% vs 8.2%, *p* = 0.094).

**Table 3:** The comparison of operative parameter between phase 1 + 2 and phase 3

	Phase 1 + 2 (*n* = 51)	Phase 3 (*n* = 49)	*P* value
Operation time (min)	175 ± 5	149 ± 5	< 0.001
Amount of bleeding (ml) (median, IQR)	5 (5–10)	5 (5–10)	0.53
Tumor size (cm)	16.4 ± 6.1	17.3 ± 7.8	0.55
Complexity (Simple/Complex)	24/27	31/18	0.11
Conversion	1 (2.0)	0 (0)	0.23
Drainage period (days) (median, IQR)	3.5 ± 4.1	3.0 ± 1.9	0.23
Complication (%)	10 (19.6)	4 (8.2)	0.094

*BMI* Body mass index, *IQR* Interqurtile range

**Fig. 3:**
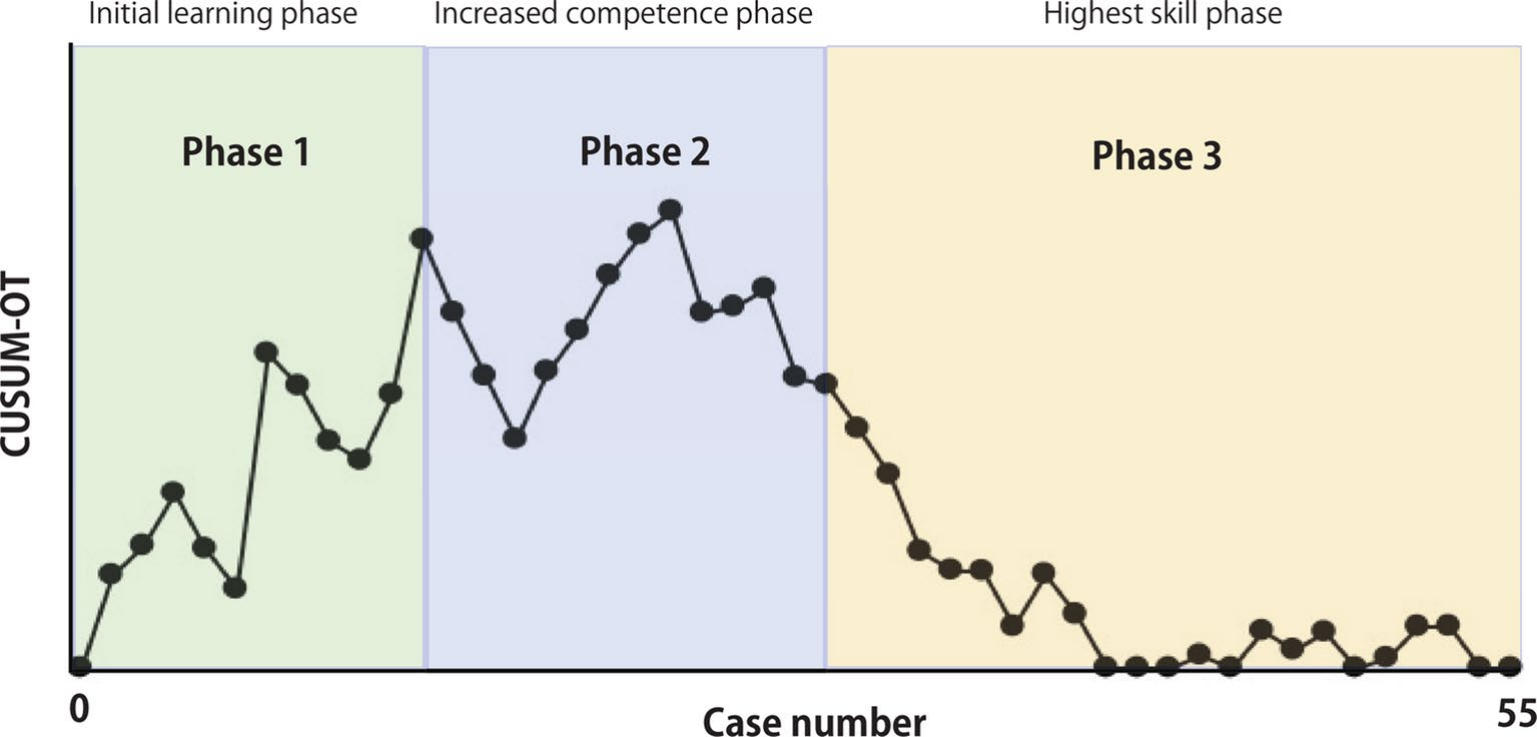
Three phases of operative time in terms of the CUSUM learning curve in the patients who underwent simple segmentectomy. CUSUM: cumulative sum

**Fig. 4:**
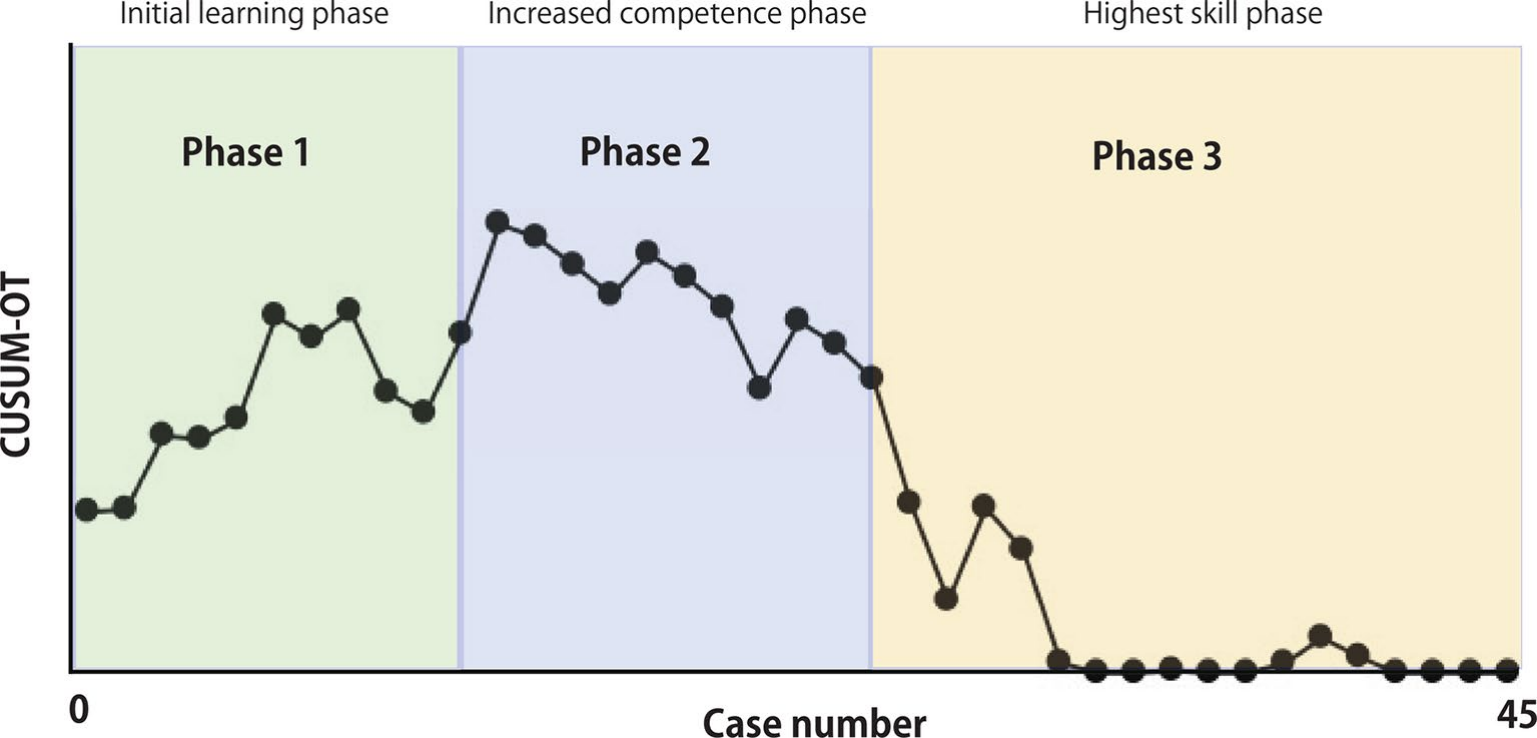
Three phases of operative time in terms of the CUSUM learning curve in the patients who underwent complex segmentectomy. CUSUM: cumulative sum

Furthermore, we examined the learning curve according to procedural complexity (simple vs. complex segmentectomy). Figures 3 and 4 show the learning curves for simple and complex segmentectomies, respectively. In simple segmentectomy, phase 1 occurred in the initial 11 cases, phase 2 occurred in the middle 12–25 cases, and phase 3 occurred in the final 26–55 cases. The inflection point was observed at 25 patients. In complex segmentectomy, phase 1 occurred in the initial 11 cases, phase 2 in the middle 12–22 cases, and phase 3 in the final 23–45 cases. The inflection point was observed at 22 patients. These results indicate that robotic complex segmentectomy requires the same number of cases to achieve the same degree of competence as simple segmentectomy.

## Discussion

This is a report pertaining to the learning curve of RATS based on our consoler- and bedside surgeon-fused approach. We documented the cases required to achieve technical competency for this procedure and compared them according to the procedural difficulty.

JCOG0802/WJOG4607L and CALGB140503 provided evidence supporting sublobar resection in non-small-cell lung cancer (NSCLC) with a tumor size ≤ 2 cm in diameter, leading to its increased adoption among thoracic surgeons [8, 9]. Furthermore, even if the tumor diameter exceeds 2 cm, segmentectomy should be indicated as a standard therapy for ground-opacity-dominant NSCLC with a tumor size ≤ 3 cm in diameter [12]. Considering that the indications for segmentectomy are expected to increase in the near future, thoracic surgeons should be familiar with this procedure.

Minimally invasive techniques for lung cancer surgery have revolutionized thoracic surgery, and robotic approaches have become increasingly popular. RATS allows a clear three-dimensional view, enabling console surgeons to spatially comprehend pulmonary anatomy. This technology enhances maneuverability and adaptability, allowing console surgeons to approach at any angle with precision. However, segmentectomy requires meticulous handling of the peripheral pulmonary vasculature and the bronchi. RATS has the advantage when it comes to performing segmentectomy because the segmental small vasculature and bronchi are easily exposed on magnified three-dimensional vision and multidirectionally dissected from the flexibly movable articulated robotic instrument. In addition, when identifying the intersegmental plane, systemic installation of ICG has been used to delineate the demarcation line on a fluorescent endoscope, which has already been implemented in firefly mode since Da Vinci X and Xi. In particular, when performing complex segmentectomy, an accurate understanding of the individual anatomical differences in the pulmonary vasculature and bronchi is important for procedural accuracy and surgical confidence. Therefore, 3D-CT reconstruction software programs enable surgeons to accurately visualize the anatomy before and during surgery through virtual simulations.

Through the TilePro DVI input, a function of direct visualization in a picture-on-picture fashion on the console screen, console surgeons can simultaneously view actual endoscopic and 3D-CT views. Therefore, RATS has a high affinity for segmentectomy in terms of both functional advantages and procedural maneuverability [13]. However, to date, few studies have documented the learning curve for this procedure [14, 15].

Zhang et al. demonstrated that technical competency for ensuring feasible perioperative outcomes was achieved in phase II at the 46th operation, which is consistent with our results demonstrating that 51 cases were required to achieve technical competence [14]. Given that RATS lobectomy required 60 cases of technical competence in our institution (data not shown), surgeons became proficient in segmentectomy faster than in lobectomy, although RATS segmentectomy was introduced slightly after RATS lobectomy. In addition, complex segmentectomy requires greater peripheral isolation simple segmentectomy, division of suitable segmental vasculature and bronchi, and the construction of several intersegmental planes. Consequently, the median OT for complex segmentectomies is substantially longer than that for simple segmentectomies. However, our initial comparison of the learning curves for simple and complex segmentectomies demonstrated that complex segmentectomies required the same number of cases as simple segmentectomies to achieve this level of competence. These results indicate that the advantages of RATS, such as TilePro, Firefly mode with ICG administration, and the use of multidirectional articulated robotic instruments, might facilitate shorter proficiency periods even in the case of complex segmentectomy.

In our fused RATS approach, the bedside surgeon can use curved suction or a cotton swab to expand the operative view, and a mechanical stapler or energy device to dissect the vasculature through the access window or robotic port, which could help novice bedside surgeons improve their surgical skills and efficiency [6]. In contrast, leading bedside surgeons can assist young console surgeons in smoothly navigating robotic surgery through easy access to the operative field. Consequently, we encountered only one case (1%) that was converted to a conventional video-assisted thoracoscopic approach due to massive bleeding from A6 during left S6 segmentectomy. Therefore, the lessons learned from analyzing the learning curve in our robotic surgical fashion will contribute to the establishment and development of straightforward robotic surgical teamwork.

Several limitations associated with the present study warrant mention. First, it was retrospective and included only a single institution with a small number of enrolled patients. Second, we did not analyze each surgeon's learning curve, instead evaluating our team's operative procedure, including all console and bedside surgeons in our department. Third, we focused only on surgical outcomes and did not evaluate the functional advantage of segmentectomy, which we assumed would be advantageous over lobectomy. Further analyses of cost-effectiveness and long-term oncological outcomes are required.

In conclusion, the data suggest that the inflection point of the learning curve was achieved after 51 robotic and manual fused segmentectomies and that complex segmentectomy yielded the same results as simple segmentectomy in achieving this level of competence. These results can be used as a guide for individual surgeons to assume their own level of proficiency regardless of procedural complexity (simple or complex segmentectomy) and to consider the composition of the team. To develop this approach further, surgical teams should consider the results of these findings.

## Data Availability

The data underlying this article will be shared upon reasonable request by the corresponding authors.

## References

[1] Louie BE, Wilson JL, Kim S, Cerfolio RJ, Park BJ, Farivar AS, et al. Comparison of video-assisted thoracoscopic surgery and robotic approaches for clinical stage I and stage II non-small cell lung cancer using the society of thoracic surgeons database. Ann Thorac Surg. 2016;102:917–24. 10.1016/j.athoracsur.2016.03.032. 27209613 10.1016/j.athoracsur.2016.03.032 PMC5198574

[2] Nakamura H, Suda T, Ikeda N, Okada M, Date H, Oda M, et al. Initial results of robot-assisted thoracoscopic surgery in japan. Gen Thorac Cardiovasc Surg. 2014;62:720–5. 10.1007/s11748-014-0441-7. 25467061 10.1007/s11748-014-0441-7

[3] Adams RD, Bolton WD, Stephenson JE, Henry G, Robbins ET, Sommers E. Initial multicenter community robotic lobectomy experience: Comparisons to a national database. Ann Thorac Surg. 2014. 10.1016/j.athoracsur.2014.02.043. 24726600 10.1016/j.athoracsur.2014.02.043

[4] Jang HJ, Lee HS, Park SY, Zo JI. Comparison of the early robot-assisted lobectomy experience to video-assisted thoracic surgery lobectomy for lung cancer: A single-institution case series matching study. Innovations (Phila). 2011;6:305–10. 10.1097/IMI.0b013e3182378b4c. 22436706 10.1097/IMI.0b013e3182378b4c

[5] Mattioni G, Palleschi A, Mendogni P, Tosi D. Approaches and outcomes of robotic-assisted thoracic surgery (rats) for lung cancer: A narrative review. J Robot Surg. 2023;17:797–809. 10.1007/s11701-022-01512-8. 36542242 10.1007/s11701-022-01512-8 PMC10209319

[6] Tane S, Tanaka Y, Nishikubo M, Doi T, Hokka D, Maniwa Y. Console and bedside surgeon fused robot-assisted thoracic surgery. Gen Thorac Cardiovasc Surg. 2023;71:730–2. 10.1007/s11748-023-01964-1. 37525063 10.1007/s11748-023-01964-1

[7] Tanaka Y, Tane S, Doi T, Mitsui S, Nishikubo M, Hokka D, et al. Factors affecting the short-term outcomes of robotic-assisted thoracoscopic surgery for lung cancer. Surg Today. 2024. 10.1007/s00595-024-02797-y. 38334800 10.1007/s00595-024-02797-y

[8] Altorki N, Wang X, Kozono D, Watt C, Landrenau R, Wigle D, et al. Lobar or sublobar resection for peripheral stage ia non-small-cell lung cancer. N Engl J Med. 2023;388:489–98. 10.1056/NEJMoa2212083. 36780674 10.1056/NEJMoa2212083 PMC10036605

[9] Saji H, Okada M, Tsuboi M, Nakajima R, Suzuki K, Aokage K, et al. Segmentectomy versus lobectomy in small-sized peripheral non-small-cell lung cancer (jcog0802/wjog4607l): A multicentre, open-label, phase 3, randomized, controlled, non-inferiority trial. Lancet. 2022;399:1607–17. 10.1016/S0140-6736(21)02333-3. 35461558 10.1016/S0140-6736(21)02333-3

[10] Wohl H. The cusum plot: Its utility in the analysis of clinical data. N Engl J Med. 1977;296:1044–5. 10.1056/NEJM197705052961806. 846547 10.1056/NEJM197705052961806

[11] Handa Y, Tsutani Y, Mimae T, Tasaki T, Miyata Y, Okada M. Surgical outcomes of complex versus simple segmentectomy for stage I non-small cell lung cancer. Ann Thorac Surg. 2019;107:1032–9. 10.1016/j.athoracsur.2018.11.018. 30550801 10.1016/j.athoracsur.2018.11.018

[12] Aokage K, Suzuki K, Saji H, Wakabayashi M, Kataoka T, Sekino Y, et al. Segmentectomy for ground-glass-dominant lung cancer with a tumour diameter of 3 cm or less including ground-glass opacity (jcog1211): A multicentre, single-arm, confirmatory, phase 3 trial. Lancet Respir Med. 2023;11:540–9. 10.1016/S2213-2600(23)00041-3. 36893780 10.1016/S2213-2600(23)00041-3

[13] Haruki T, Kubouchi Y, Kidokoro Y, Matsui S, Ohno T, Kojima S, et al. A comparative study of robot-assisted thoracoscopic surgery and conventional approaches for short-term outcomes of anatomical segmentectomy. Gen Thorac Cardiovasc Surg. 2024;72:338–45. 10.1007/s11748-023-01983-y. 37934374 10.1007/s11748-023-01983-y PMC11018688

[14] Zhang Y, Liu S, Han Y, Xiang J, Cerfolio RJ, Li H. Robotic anatomical segmentectomy: An analysis of the learning curve. Ann Thorac Surg. 2019;107:1515–22. 10.1016/j.athoracsur.2018.11.041. 30578780 10.1016/j.athoracsur.2018.11.041

[15] Igai H, Numajiri K, Ohsawa F, Nii K, Kamiyoshihara M. Comparison of the learning curve between uniportal and robotic thoracoscopic approaches in pulmonary segmentectomy during the implementation period using cumulative sum analysis. Cancers (Basel). 2023. 10.3390/cancers16010184. 38201611 10.3390/cancers16010184 PMC10778519

